# Medical News and Miscellany

**Published:** 1899-09

**Authors:** 


### Medical News and Miscellany.

**Dr. J. A. Boyd** of Thorndale, Texas, died Aug. 11 (ult).

**Practice for Sale.**—$650 will buy a good house, barn, etc., and a $2000 yearly practice, monopoly—no competition, in a desirable section. Address, Dr. J. E. Standifer, Eolian, Stephens County, Texas.

**Dr. W. J. Miller,** of McGregor, died at his home in that place on July 16, 1899.

---

**For Sale.**—A good practice and location in South Texas; good reasons for selling. Address Dr. Ed., care TEXAS MEDICAL JOURNAL.

---

**Change of Address.**—Dr. F. P. Kennedy, from Dublin to Topaz; Dr. Stirling Price, from Richland to Powell, Texas; Dr. W. P. Perkins, from Beaumont, Texas, to Leesville, La.; Dr. E. E. Scott, Deming's Bridge to Hawley, Texas.

---

**The New Orleans Polyclinic,** thirteenth annual session, opens November 20, 1899; closes May 10, 1900. Every inducement in clinical facilities for those attending. The specialties are fully taught. Further information, New Orleans Polyclinic, New Orleans, La.

---

**Attention, Doctor.**—Fine location—no opposition; thickly settled community; black land. $400 takes practice, nice dwelling, lot, barn, office and a small supply of drugs. Going to retire from practice. This is a good bargain. If you mean business write at once. Address Dr. G. B., care TEXAS MEDICAL JOURNAL.

---

**To the Mississippi Valley Medical Association Meeting in Chicago.**—Are you going? If not you will miss one of the best meetings and one of the "best times" of your life. Better decide to go, Doctor, and get the benefit of the meeting, stay a while in Chicago and see what the doctors there are doing in hospital work. Don't forget that we want to bring the 1900 meeting to Texas.

---

**Chestnut Leaves for Whooping Cough.**—Dr. F. A. Remley, of Alvin, Texas, writes to the JOURNAL as follows: "I want to say through the TEXAS MEDICAL JOURNAL to the physicians of Texas: Try giving whooping cough patients either a reliable fluid extract of chestnut leaves, or get the fresh leaves and make a tea and give this. I usually give from 20 to 60 drops of the fluid extract of chestnut leaves four or five times a day, allowing the patient nothing to drink for half hour after taking; or make a decoction of leaves and give teaspoon doses. This for children one year old or older. Have never had it fail to cure in six days.'

**Doctor.**—When making your arrangements to attend the meeting of the Mississippi Valley Medical Association, it will be to your interest to remember that the "Iron Mountain" and the "Wabash" will make special rates, and then, such elegant equipments and services! The "Wabash" is one the best equipped roads in Uncle Sam's dominion, and it can carry you from St. Louis to Chicago in the shortest possible time and with the greatest comfort to you.

---

**Low Rate Excursions to Mexico.**—September 7th to 12th, inclusive, excursion tickets from all points in Texas will be sold account National Fiestas (celebration of Mexico's Independence) at extremely low rates, via Laredo and the Mexican National Railroad.

For full particulars, call on nearest ticket agent, or address:

E. Muenzenberger,
Com. Agt., Mex. National R. R. Co.,
San Antonio, Texas.

---

**Courier of Medicine, St. Louis.**—We are pleased to note that the publication of this sterling periodical, which was suspended some time ago, has been resumed. The July number has been received. It is fully up to the standard adopted by its former management, when it began its career in 1879. The new management is C. R. Dudley, M. D., editor-in-chief; Drs. Joseph Grindon, E. F. Smith and W. A. Shoemaker, associate editors. It is published by the Courier Medicine Company, under the auspices of the Library Association of St. Louis. Terms, $2 a year.

---

### Appendicitis Does Not Always Need the Knife.

---

"I protest against the use of opium, except in rare cases, as it has a tendency to mask the symptoms of the disease and leads the patient to the grave. I protest against the argument of Dr. Niles, that every case ought to be operated upon, and the appendix is never to be left. Out of 300 post-mortems on as many bodies, it was found that 100 of the individuals had had appendicitis at some time in their lives, and had all recovered from the disease. I dispute the assertion that through surgical operations all but two per cent. of the cases can be saved. I challenge any operator in the room to take 100 well persons and operate upon them without killing more than two per cent. We all fail, gentlemen. I do not know why, but we all fail. I do not believe in operating on all cases of appendicitis. I'd rather have a live man with an appendix than a dead one without one. (Applause). I do not believe with the witty Frenchman that no case is complete without an autopsy. (Laughter). If the patient is no worse after forty-eight hours of observation, let him alone; let him get well."—*Dr. W. W. Keen, at the Denver Meeting of the American Medical Association.*

