# Erratum

**Published:** 1899-10

**Authors:** 


**Erratum.**—In our last we said Dr. Sterling Price had removed from Richland to Powell, Tex. It should have been Dr. Jno. W. Price; Dr. Sterling Price is still at Barry, Tex.

